# Quantifying iris pigmentation irregularities following an avian influenza outbreak: implications for population-level disease surveillance in a colonial seabird

**DOI:** 10.1093/conphys/coag069

**Published:** 2026-09-24

**Authors:** Christina Petalas, Jolene A Giacinti, Jean-Francois Rail, Raphaël A Lavoie, Jennifer F Provencher, Stephanie Avery-Gomm, Kyle H Elliott, Yannick Seyer

**Affiliations:** Department of Natural Resource Sciences, Macdonald-Stewart Building, McGill University (Macdonald Campus), 21111 Lakeshore Road, Sainte-Anne-de-Bellevue, Québec, H9X 3V9, Canada; Science and Technology Branch, Environment and Climate Change Canada, Canada Centre for Inland Waters, 867 Lakeshore Road, Burlington, Ontario, L7S 1A1, Canada; Canadian Wildlife Service, Environment and Climate Change Canada, Suite 801, 1550 d'Estimauville Avenue, Québec, Quebec, G1J 0C3, Canada; Science and Technology Branch, Environment and Climate Change Canada, Suite 801, 1550 d'Estimauville Avenue, Québec, Quebec, G1J 0C3, Canada; Science and Technology Branch, Environment and Climate Change Canada, National Wildlife Research Centre, Carleton University, 1125 Colonel By Drive, Ottawa, Ontario, K1A 0H3, Canada; Science and Technology Branch, Environment and Climate Change Canada, 335 River Road, Ottawa, Ontario, K1A 0H3, Canada; Department of Natural Resource Sciences, Macdonald-Stewart Building, McGill University (Macdonald Campus), 21111 Lakeshore Road, Sainte-Anne-de-Bellevue, Québec, H9X 3V9, Canada; Canadian Wildlife Service, Environment and Climate Change Canada, Suite 801, 1550 d'Estimauville Avenue, Québec, Quebec, G1J 0C3, Canada

**Keywords:** Abnormal iris pigmentation, antibodies, avian influenza virus, northern gannet, population monitoring, sublethal effects, wildlife health

## Abstract

Emerging infectious diseases can have catastrophic impacts on wildlife populations, yet identifying individuals that survived exposure, especially when external symptoms are absent, remains challenging. Since 2021, a virulent strain of highly pathogenic avian influenza virus (HPAIV; H5N1 clade 2.3.4.4b) has caused unprecedented mortality in wild birds. Northern gannets (*Morus bassanus*) suffered dramatic mortality and reproductive failure at North America’s largest colony in 2022. Following the outbreak, Lane *et al.* reported that a subset of gannets at a UK colony displayed darkened irises associated with HPAIV seropositivity. Here, we build on this work by quantifying iris pigmentation irregularity and evaluating whether greater irregularity predicts prior exposure, measured by anti-nucleoprotein (NP) and anti-hemagglutinin (H5) antibodies with standardized field-based photography. Iris pigmentation was strongly associated (*ρ* = −0.72) with NP antibody reactivity (S/N ratios), and the probability of NP seropositivity increased with irregularity (50% at 40% irregularity, 65% at 50%, and >90% above ~77%). We observed weaker positive associations with H5 antibody reactivity (percent inhibition, PI%; *ρ* = 0.30), consistent with differences in antibody persistence and detection dynamics. Among NP-seropositive individuals, NP antibody reactivity did not vary with iris irregularity, whereas H5 antibody reactivity increased with pigmentation extent. Pre-outbreak data defined a narrow range of iris pigmentation irregularity (≤8%), supporting a threshold separating baseline from elevated values. Our findings support earlier evidence that iris pigmentation irregularities reflect prior HPAIV exposure and demonstrate that quantifying their extent provides greater resolution for population-level monitoring. Iris scoring offers a rapid, minimally invasive approach for estimating prior exposure in post-outbreak contexts, particularly where serological sampling is limited. Rather than serving as a diagnostic proxy for individual exposure history, this metric provides an epidemiological indicator when interpreted alongside serological and ecological data. Future research should determine whether iris pigmentation remains stable, fades, progresses or changes, following subsequent HPAIV exposures.

## Introduction

Avian influenza viruses (AIVs) have circulated in some species of wild birds for over a century (Hoye *et al.*, 2010; Lycett *et al.*, 2019), but recent decades have seen increases in the frequency and severity of detected outbreaks (Stallknecht and Brown, 2008; Chatziprodromidou *et al.*, 2018; Blagodatski *et al.*, 2021). Since 2021, a particularly virulent strain of highly pathogenic avian influenza virus (HPAIV; H5N1 clade 2.3.4.4b) has caused unprecedented global mortality in wild birds and poultry (Camphuysen *et al.*, 2022; Wille and Barr, 2022). Seabirds have been heavily affected, likely due to factors such as limited prior immunity, dense nesting conditions and high rates of direct contact, all of which facilitate viral transmission (Lang *et al.*, 2016; Dewar *et al.*, 2023; McPhail *et al.*, 2024). Mass die-offs due to HPAIV have been documented at several seabird breeding colonies, with some populations experiencing losses exceeding 70% (Banyard *et al.*, 2022; Camphuysen *et al.*, 2022; Rijks *et al.*, 2022; Pohlmann *et al.*, 2023; Knief *et al.*, 2024; Tremlett *et al.*, 2024). HPAIV has therefore emerged as a major conservation concern for some seabird species, contributing to one of the largest documented wild bird mortality events associated with avian influenza (CMS FAO Co-convened Scientific Task Force on Avian Influenza and Wild Birds, 2022).

Northern gannets (*Morus bassanus*) have been among the most severely impacted seabird species, with large-scale mortality events reported at 40 of 53 global colonies in 2022, including five of six Canadian colonies (Giralt Paradell *et al.*, 2023; Seyer and Guillemette, 2023; Avery-Gomm *et al.*, 2024; Lane *et al.*, 2024; Giacinti *et al.*, 2024b). Their dense colonial nesting conditions, combined with limited prior exposure to AIV (McLaughlin *et al.*, 2025), likely facilitated rapid viral spread (McPhail *et al.*, 2024). The widespread mortality observed across the North Atlantic during the 2022 breeding season has raised urgent questions about colony-level vulnerability, immunity, and the potential for repeated outbreaks. Although large outbreaks were not widely reported in 2023, serological evidence indicates prior exposure to HPAIV in seabirds in Atlantic Canada (McLaughlin *et al.*, 2025), suggesting persistent circulation of this virus. Long-term monitoring has documented cumulative environmental stressors for gannets, including prey depletion, ocean warming, and anthropogenic activities (Guillemette *et al.*, 2018; Grémillet *et al.*, 2020; Montevecchi *et al.*, 2021). Together, these pressures underscore the importance of understanding both short-term survival following HPAIV outbreaks and the longer-term recovery potential of gannet populations. Developing reliable, minimally invasive indicators of prior infection that operate at the population-level is therefore a priority for post-outbreak seabird monitoring, as they enable estimation of the proportion of individuals exposed during outbreak events and the persistence of survivors within the breeding population. This information is critical for linking exposure history to subsequent changes in survival, breeding participation, and population recovery trajectories.

While direct mortality has dominated attention, emerging evidence suggests HPAIV may also be associated with sublethal effects in surviving seabirds, including altered movement patterns, reproductive variation and physiological changes; however, reported effects are heterogeneous across colonies and contexts (Duriez *et al.*, 2023; Careen *et al.*, 2024; Lewis *et al.*, 2025; Ponchon *et al.*, 2026). Identifying individuals with prior HPAIV exposure, however, remains difficult, particularly in remote colonies where capture and blood sampling are costly and logistically demanding and may increase disturbance to breeding birds. This has prompted interest in developing minimally invasive field tools that can distinguish previously exposed individuals from unexposed individuals without reliance on invasive blood sampling, thereby supporting monitoring of colony recovery and resilience following mass mortality events (Nalepa *et al.*, 2024).

One promising field indicator is the recently documented occurrence of abnormal iris darkening in surviving gannets following HPAIV outbreaks, in which portions or all of the normally pale-blue iris become blackened (Lane *et al.*, 2024). Lane *et al.* (2024) first highlighted this trait as a minimally invasive indicator of prior HPAIV exposure, reporting that individuals with qualitatively darkened irises were more likely to be H5-seropositive shortly after the 2022 outbreak. Building on this, Lewis *et al.* (2025) further demonstrated that gannets with irregular iris pigmentation showed comparable breeding success to unaffected individuals, supporting the use of iris pigmentation as a viable post-outbreak population monitoring metric.

While gannet irises are naturally pale-blue, recent ophthalmological examinations indicate that abnormal iris pigmentation in gannets is frequently accompanied by signs of uveitis and increased melanin deposition within the iris, providing evidence that both inflammatory processes and melanocytic changes may contribute to the observed darkening (Olson and Owens, 2005; Toomey *et al.*, 2010; Corbett *et al.*, 2024; Fontaine *et al.*, 2026). Fontaine *et al.* (2026) further found that abnormal iris pigmentation was associated with H5 seropositivity, supporting a link between these ocular changes and prior HPAIV exposure. Although the functional and long-term consequences of these alterations remain uncertain, these findings provide a mechanistic basis for considering iris pigmentation irregularities as a potential indicator of prior HPAIV exposure. Whether this response is specific to HPAIV exposure or indicative of a broader inflammatory response to infection remains unresolved. Ocular changes have been reported in some birds following HPAIV infection. For example, Alexandrou *et al.* (2025) documented cloudy corneas in Dalmatian pelicans (*Pelecanus crispus*) with H5-specific antibodies, and experimental infections have induced corneal opacity in ducks (Brown *et al.*, 2008; Yamamoto *et al.*, 2016). These observations indicate that HPAIV can induce ocular changes, including alterations in pigmentation and corneal appearance, although the specific manifestations and prevalence likely vary among species. To our knowledge, such pronounced and visible post-infection iris darkening has not been reported in other avian species, making this a potentially unusual phenotypic response to viral infection. This apparent uniqueness may enhance its value as a field-based indicator for monitoring post-HPAIV exposure in this species.

If reliably associated with AIV seropositivity, iris pigmentation irregularities could serve as a rapid, practical screening tool to estimate the proportion of previously exposed individuals within a population. At the colony-level, repeated assessments of iris pigmentation could provide snapshots of infection history over time, offering a means to monitor population-level post-outbreak survival, even when individual birds are unmarked. Linking these data with reproductive metrics, such as breeding success and chick survival, would further allow evaluation of how prior AIV exposure affects population resilience, integrating minimally invasive health surveillance with demographic monitoring.

Despite these advances, the relationship between the degree of iris pigmentation irregularity and serological evidence of prior AIV exposure has not been empirically validated. Previous work has largely relied on categorical classification of eye colour (Lane *et al.*, 2024), and no study has quantitatively evaluated whether variation in pigmentation extent corresponds to antibody-based measures of past infection, nor evaluated how this relationship may vary among AIV antibody targets. This represents a critical gap in evaluating the reliability and practical application of iris pigmentation as a minimally invasive, population-level monitoring metric. If informative thresholds can be identified, practitioners could use iris scoring to improve estimates of prior exposure, identify heterogeneity within colonies, and track post-outbreak recovery trends when serological sampling is logistically constrained. Addressing this question is therefore relevant for monitoring the longer-term impacts of HPAIV and informing conservation management.

Here, we combine serological and photographic data to evaluate whether iris pigmentation irregularities are reliably associated with antibody-based evidence of past AIV exposure. Using whole blood collected on filter strips, an efficient, field-adaptable method for serological sampling (Giacinti *et al.*, 2025), we tested for two AIV antibody targets [anti-nucleoprotein (NP) and anti-hemagglutinin (H5) antibodies], which provide evidence of past exposure to AIV in wild birds (Giacinti *et al.*, 2025). We also developed a standardized method to quantify iris pigmentation irregularities from digital photographs and compared Enzyme-Linked Immunosorbent Assay (ELISA)-derived serological antibody reactivity [NP: sample-to-negative (S/N) ratios; H5: percent inhibition (PI%) values] between gannets with and without visible irregularities during the 2023 breeding season.

We hypothesize that increasing iris pigmentation irregularity is associated with a higher likelihood of serological evidence of prior AIV exposure, reflecting differences in exposure and immune response following infection. Specifically, we predict that individuals with more irregular (darker or more heterogeneous) irises would be more likely to test positive for anti-NP and anti-H5 antibodies based on established thresholds, thereby supporting the use of pigmentation extent as a graded indicator of prior exposure likelihood. Additionally, we tested whether antibody reactivity was associated with body mass, used as a proxy for physiological condition, given evidence that viral infection can reduce condition in seabirds (Siebert *et al.*, 2012). This analysis aimed to assess whether this proxy of physiological condition covaried with iris pigmentation irregularities. Finally, we explored whether repeated photographic assessments revealed temporal variability in iris irregularities across a breeding season, as a preliminary evaluation of short-term individual stability. Collectively, these analyses aim to further assess the potential of iris pigmentation as a practical, low-cost field metric of prior AIV exposure, supporting minimally invasive monitoring of disease impacts on wild seabird populations.

## Materials and methods

### Sample collection

**Figure 1:**
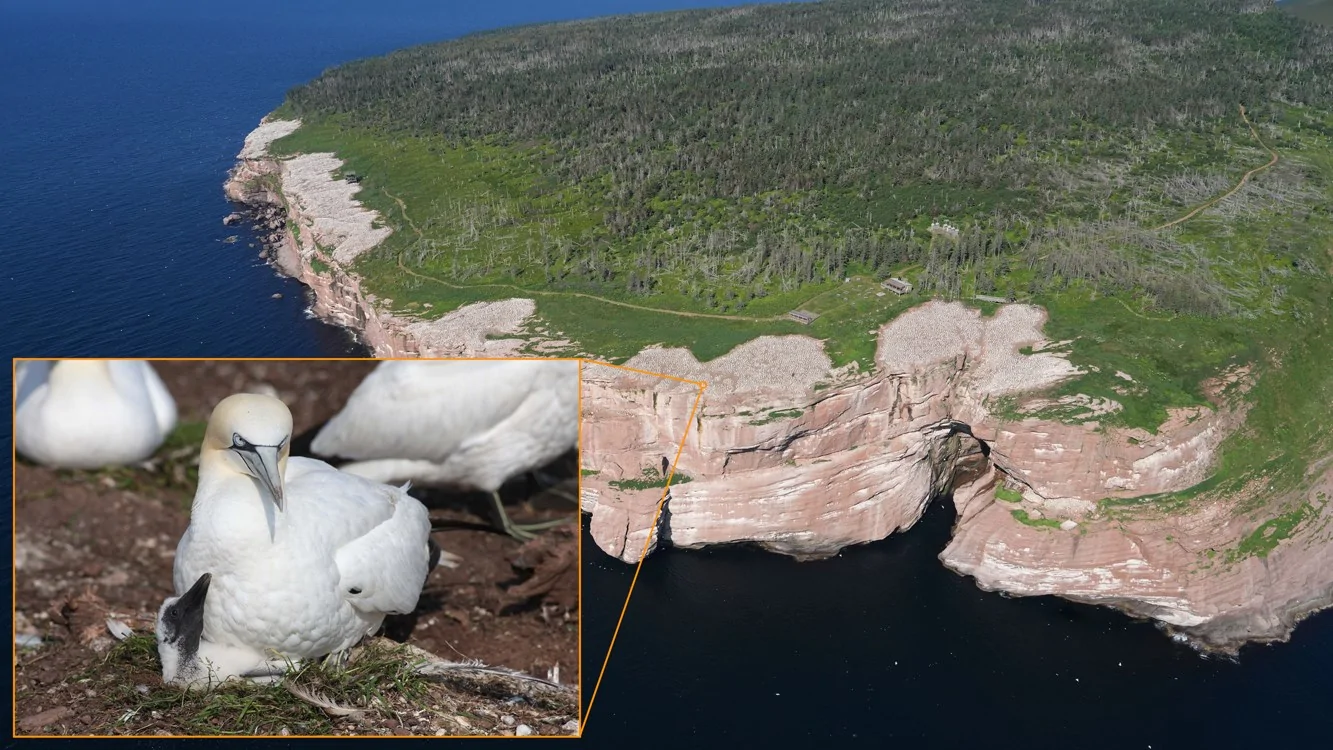
Northern gannet (*M. bassanus*) breeding colony at Bonaventure Island, Québec. The inset shows an adult gannet with its chick at the nest. Photographs by Jean-Francois Rail.

Canada supports the entire North American breeding population of northern gannets and, prior to the 2022 HPAIV outbreak, hosted ~13% of the global population (Mowbray, 2020). This included the large Bonaventure Island colony (~104 000 breeding individuals; Rail, 2021) in Québec, Canada (48.4943°N, 64.1608°W), located in the Gulf of St. Lawrence. Bonaventure Island is located within Parc national de l’Île-Bonaventure-et-du-Rocher-Percé and is protected under both provincial jurisdiction (as a park managed by the Société des établissements de plein air du Québec; Sépaq) and federal jurisdiction (as a Migratory Bird Sanctuary established under the Migratory Birds Convention Act and associated regulations, administered by Environment and Climate Change Canada (ECCC)). Gannets breed on the south-east side of the island on cliffs or on the plateau where the majority of pairs nest (Fig. 1). On the plateau, nests are regularly spaced ~60–80 cm apart (Poulin, 1968). From 11 to 14 September 2023, during the breeding season, 65 adult gannets were captured on or near their nests at the Bonaventure Island colony.

Breeding gannets were captured across the plateau area at the outer edge of the colony while on or near their nest using a noose-pole. Individuals were targeted based on iris pigmentation, attempting to sample both birds with irregular and regular iris pigmentation. Of the 30 individuals targeted for irregular iris pigmentation, 13% were captured on nests with chicks; the remaining birds were either on empty nests (47%) or not on a nest at the time of capture (40%). In contrast, 80% of the 35 individuals with regular iris pigmentation were captured on active nests with chicks, 17% on an empty nest and 3% not on a nest. All individuals appeared in good body condition and exhibited no overt behavioural impairments. Individuals were observed engaging in routine breeding activities (e.g. nest attendance, partner exchange, or chick provisioning) at the time of sampling. In addition to the methods described below, all captured birds were marked with a United States Geological Survey (USGS) metal band. All live bird sampling followed approved animal use protocols, applicable federal and provincial wildlife permits and safe work procedures obtained by ECCC: Banding Permit 10711 B, Animal Use Protocol 23JFR01, Migratory Bird Sanctuary Permit RE-8 and Scientific Permit SC-1570. Research was also conducted under the Memorandum of Understanding between Sépaq and ECCC.

The tarsal vein was pricked using a 24-gauge needle. Approximately 3–4 drops of whole blood (~0.1 ml) were collected from each bird directly from the vein onto a Nobuto blood filter strip (Advantec MFS, Inc., USA; Product #49010010), fully saturating the strip following the procedure described in Giacinti *et al.* (2025). After sampling, all filter strips were air-dried in tented paper coin envelopes for at least 24 h and preserved at room temperature. Oropharyngeal and cloacal swabs were also taken from each bird to test for AIV infection. The oral and cloacal swabs were placed into a single vial of 3 ml Universal Transport Medium (Copan Italia S.p.A., Brescia, Italy) and represent a single sample per individual. Each tube was immediately placed in a freezer bag for temporary storage during daily field activities and stored at −20°C within a few hours until they could be transported to the laboratory for avian influenza testing.

During the handling of adult gannets, high-resolution photographs of each individual’s left and right eyes were captured using a handheld smartphone camera (Samsung Galaxy S22; model SM-S901W). Images were taken at ~20 cm from the bird under natural light conditions, using autofocus, no flash and default camera settings (f/1.8, 23 mm equivalent focal length). Date and time information were recorded for each photograph. Gannets were restrained by an experienced handler using a standardized handling technique, with the bill gently secured to prevent head movement and the body and wings supported against the handler’s torso, following established seabird handling protocols to minimize stress and movement during measurements and photography. Photographs were collected under natural light conditions between ~11:00 and 14:00 across all four sampling days to standardize illumination. Sampling was restricted to clear or lightly overcast conditions, and birds were oriented to minimize glare and shadows, ensuring consistent visibility of pupil diameter and iris pigmentation. The camera angle relative to the eye was kept as perpendicular as possible to reduce distortion, although minor variation in angle and distance was unavoidable in field conditions. Prior to release, the body mass of each bird was measured using a 4000-g Pesola scale (10 g precision).

To provide important context for interpreting iris pigmentation irregularities as a potential biomarker, we conducted a preliminary assessment of within-individual temporal stability. A subset of seven adult gannets with clearly identifiable iris irregularities was photographed on two to three occasions during the 2024 breeding season. Individuals were confirmed and identified based on nest location within the colony and USGS metal band numbers. Birds were first documented during incubation and subsequently re-photographed opportunistically during chick-rearing over a period of ~6–8 weeks. Images were obtained from distances of 2–5 m using a DSLR camera fitted with a 300–400 mm telephoto lens (f/10 aperture, ISO 640, 1/1600 s shutter speed), providing sufficient resolution for detailed iris assessment without capturing or disturbing the birds. This approach was designed as a short-term, exploratory evaluation of visual consistency in pigmentation patterns under natural field conditions and was not intended to assess long-term inter-annual stability of the trait. However, the same repeated-imaging framework would be readily applicable across multiple breeding seasons where individuals are permanently marked and can be reliably re-identified.

### Sample preparation and laboratory testing

The detection of anti-NP antibodies was performed using the IDEXX AI MultiS Screen Ab test (IDEXX Canada, Product # 99-12119) at the National Wildlife Research Centre (NWRC). Nobuto filter strips saturated with whole blood were cut and eluted in 400 μl of phosphate-buffered saline (pH 7.2; GibcoTM, USA, Product # 20012027) at 4°C for 24 h (as described by Giacinti *et al.*, 2025). ELISA testing was then conducted according to the manufacturer’s instructions (Brown *et al.*, 2009). All eluates were also tested for anti-H5 antibodies at the National Centre of Foreign Animal Disease (NCFAD), using previously established methods (Hochman *et al.*, 2023). The S/N ratios and PI% values derived from these semi-quantitative ELISA assays provide relative measures of antibody reactivity to the target antigen, where lower S/N ratios and higher PI% values indicate stronger reactivity.

An optimized threshold of S/N < 0.77 was used to classify samples as positive for anti-NP antibodies, following threshold optimization performed at NWRC for frozen Nobuto strip testing as discussed in Giacinti *et al.* (2025). A PI value ≥20.37% was considered positive for H5 antibodies, based on the optimized cut-off for frozen Nobuto strips (Giacinti *et al.*, 2025). The NP ELISA detects antibodies broadly reactive to influenza A viruses and does not distinguish between highly pathogenic and low-pathogenic strains. In contrast, the H5 assay detects antibodies specific to H5 subtype AIVs, but similarly does not definitively differentiate between high- and low-pathogenicity H5 viruses.

Pooled oropharyngeal and cloacal swabs were submitted to the Animal Health Laboratory (AHL) at the University of Guelph, where total RNA was extracted using the QIAamp Viral RNA Mini Kit (Qiagen). Samples were tested using real-time reverse-transcription PCR (RT-PCR) targeting the influenza A matrix (M) gene (Nagy *et al.*, 2021) and a specific H5 assay (James *et al.*, 2022), following standardized Canadian Animal Health Surveillance Network protocols (Giacinti *et al*., 2024b). Any samples testing positive at AHL would have been forwarded to the NCFAD for confirmatory testing, including additional RT-PCR, virus isolation and sequencing. However, all samples in this study tested negative.

### Image processing

**Figure 2:**
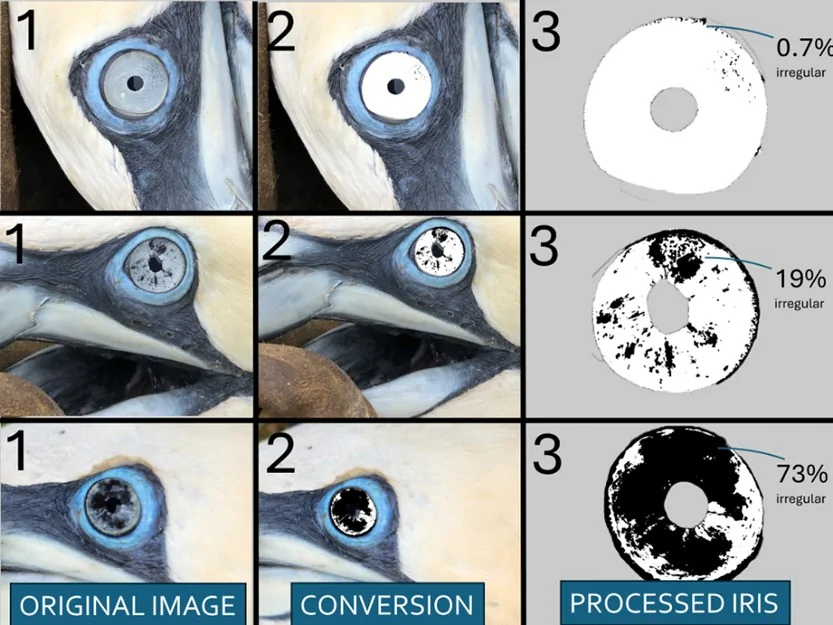
Example of image processing procedure where northern gannet photographs taken in the field (step 1) are analysed using Photoshop (step 2) to isolate the iris and then calculate the percentage of irregularities in each iris (step 3).

All eye images were processed manually by a single trained observer to ensure reproducibility and consistency using Adobe Photoshop version 25.4. Each image was first cropped to isolate the iris, excluding the pupil and sclera. If necessary, global brightness was adjusted uniformly across the entire image using the ‘Brightness/Contrast’ tool to correct for minor under- or over-exposure and improve visibility of iris boundaries. Adjustments were applied equally to all colour channels and did not involve localized editing, thresholding, alteration of individual pixel values, or contrast between dark and light regions of the iris. All quantification of iris pigmentation was conducted after this standardized adjustment to ensure consistent interpretation across images. The images were then converted to grayscale using the ‘Mode’ function and subsequently transformed into a binary (black-and-white) format using the ‘Threshold’ adjustment tool (Fig. 2). This resulted in only black and white pixels while removing all colour. The degree of irregular iris pigmentation was quantified by calculating the ratio of black to white pixels within the isolated iris area. This ratio, hereafter referred to as iris pigmentation irregularity (%), represents the extent of visible dark pigmentation within the iris, quantified on a scale from 0% (no irregularity) to 100% (complete darkening). Iris pigmentation irregularities were evaluated for each eye separately, and the eye exhibiting the most pronounced pigmentation irregularity (hereafter, the iris with greater irregularity) was used to assign an individual iris score.

Because the orientation of the eyeball can vary slightly during photography, resulting in an elliptical rather than perfectly circular projection of the iris, pigmentation was quantified as a proportional measure rather than as an absolute area or shape-based metric. Specifically, the degree of iris pigmentation irregularity was calculated as the ratio of black to white pixels within the isolated visible iris area, rendering the metric invariant to moderate changes in iris shape or size caused by viewing angle. To minimize angular distortion, photographs of handled birds were taken at close range (~20 cm) with the camera aligned as perpendicular as possible to the eye surface, and individuals with images showing pronounced obliquity, partial iris occlusion, or poor focus were excluded from analysis.

### Quality assurance and quality controls

To evaluate the consistency and reproducibility of iris pigmentation quantification, both within-observer and inter-observer assessments were conducted. Within-observer repeatability was assessed by randomly selecting 10% of individuals from the full dataset using a random number generator. For each selected individual, the same trained observer who processed the complete dataset independently reprocessed the original eye images using the identical workflow and software settings, blind to the initial results. The resulting estimates of iris pigmentation irregularity (%) were compared with the original measurements.

In addition, 5% of individuals were subjected to triplicate image processing to further assess measurement precision. Because traditional relative percent difference (RPD) calculations can produce inflated values when measurements are near zero, a modified RPD was calculated following Nuzzo (2018).


\begin{align*}&\mathrm{Modified}\ \mathrm{RPD}\ \left(\%\right)\\&=\!\frac{\ \left(\mid\ \mathrm{Iris}\ \mathrm{irregularities}-\mathrm{Duplicate}\ \mathrm{iris}\ \mathrm{irregularities}\mid \right)}{\left(\mathrm{Iris}\ \mathrm{Irregularities}+\mathrm{Duplicate}\ \mathrm{iris}\ \mathrm{irregularities}\right)/2+0.5}\!\! \times\!\! 100 \end{align*}

A small constant (0.5) was added to the denominator based on the distribution of observed values to prevent inflation of RPD estimates at low levels of iris pigmentation irregularity. This adjustment ensures that measurements near zero do not produce disproportionately large RPDs, providing a more stable estimate of repeatability across the full range of observed values. An RPD < 20% was considered acceptable, consistent with commonly applied quality-control thresholds for duplicate measurements (Government of British Columbia, 2016).

Within-observer repeatability was high. The mean modified RPD was 16.20 ± 17.42%, with 73.10% of values falling within the pre-established acceptable range. In addition, we calculated the absolute difference between duplicate measurements. Small absolute differences, despite potentially high RPD, were considered acceptable when they fell within a pre-established threshold (<0.5%), with an average error of 0.55 ± 3.86%, supporting consistency even when RPD values were high due to near-zero measurements. Observed minor variability between duplicates was likely associated with differences in manual delineation at the iris–pupil and iris–sclera interfaces. A linear regression model was fit to assess how much variation in the duplicate measurements could be explained by the original measurements. A strong correlation between original and duplicate values (*R*^2^ = 0.993) further confirmed measurement precision, meaning that the original measurement explained 99.3% of the variance in the duplicated measurement. This near perfect model fit suggests that repeated processing of the same images yields nearly identical results, showing that the original measurements are highly repeatable and not substantially influenced by random error or subjectivity in image processing.

Inter-observer reproducibility was assessed by having a second observer independently process a subset of the same images using only the methodological description provided in this manuscript without formal training. The observer was not provided with the original measurements, example scores, or supplementary guidance beyond the written protocol within this paper. Comparisons between observers, therefore, assessed both observer-related variation and the extent to which the image-processing workflow could be reproduced using the methods described herein. Inter-observer reproducibility was high. Independent analysis by a second observer produced measurements that differed by an average of 4.10 ± 3.75% (absolute difference), with a small mean bias of −3.53 ± 4.31%. Measurements from the two observers were strongly correlated (*R*^2^ = 0.992, *P* < 0.001), indicating that the image-processing protocol yielded highly consistent estimates when implemented by different analysts using only the methods described in this study.

To establish a baseline threshold for iris pigmentation irregularity in gannets prior to the outbreak, we processed images of gannets taken from the same Bonaventure Island colony before 2021. These archive images were taken from randomly selected individuals over multiple years without a standardized procedure. Photographs were taken using a Canon EOS 5D Mark II or Canon EOS 10D with either a 75–300 mm or 400 mm lens (f/10 aperture, ISO 640, 1/1600 s shutter speed), typically from a distance of 2–5 m from the birds, sufficient to resolve the iris without physically disturbing the individuals. All images were of sufficient resolution and clarity to allow quantification comparable to close-up photographs of handled birds. These images, spanning from 2004 to 2018, provided a reference baseline for the typical range of iris irregularities observed in the population prior to documented exposure to HPAIV (*n* = 28). We then compared this pre-outbreak distribution with post-outbreak birds that were visually assessed in the field as lacking obvious iris pigmentation abnormalities (i.e. non-black eyes) to evaluate whether these individuals fell within the historical baseline range of pigmentation irregularity.

### Statistical analysis

All analyses were conducted using R software version 4.4.2 (R Core Team, 2025). To generate a primary bird-level metric of iris pigmentation irregularity, we calculated the mean of the two iris irregularity scores (left and right), which was used as the main measure of overall iris irregularity. Additional sensitivity analyses were conducted using (i) the iris with greater irregularity and (ii) left and right eyes separately, to evaluate the robustness of results and practical field scenarios in which only one eye may be visible. To assess the relationship between iris pigmentation irregularities and antibody reactivity, we used Spearman’s rank correlation tests (ρ) using the *cor.test()* function to examine associations between iris pigment irregularities and the continuous ELISA test results: S/N ratios for anti-NP antibodies and PI% for anti-H5 antibodies. These were tested separately for anti-NP and anti-H5 antibodies. These non-parametric correlations were appropriate due to the continuous nature of the antibody measures and the non-normal distribution of iris pigment irregularity scores. All assumptions of normality and homogeneity of variances were assessed using the Shapiro–Wilk test and Levene’s test, respectively.

Initial correlation analyses included both seronegative and seropositive individuals to evaluate whether iris pigmentation irregularity captures a population-level transition from baseline (pre-exposure) phenotypes towards post-exposure phenotypes across the full spectrum of antibody responses. However, because antibody assay thresholds define biologically meaningful categories of exposure, we additionally repeated correlation analyses using seropositive individuals only (anti-NP antibody–positive or anti-H5 antibody–positive, respectively). This approach allowed us to assess whether variation in antibody assay reactivity covaried with iris pigmentation irregularity within exposed birds, consistent with our exposure-based hypothesis. These subset analyses were conducted using the mean iris irregularity score as the primary metric, with supplementary sensitivity analyses using the iris with greater irregularity and left and right irises separately to assess robustness and practical field applicability when only one eye may be visible.

Next, to assess whether iris pigment irregularity was predictive of AIV serological status, we conducted logistic regressions using the *glm()* function with a binomial error distribution. The outcome variable was the binary interpretation of the anti-NP antibody test (‘Positive’ or ‘Negative’). Iris pigment irregularity was included as the predictor in separate models: using the mean across both irises, the iris with greater irregularity, and left and right eyes separately. Parallel logistic regressions were conducted using binary anti-H5 antibody status (based on established PI% thresholds) as the outcome.

To facilitate interpretation in a monitoring context, we conducted a complementary threshold-based analysis using a biologically informed cut-off derived from pre-outbreak reference data to evaluate whether antibody reactivity differed with iris pigmentation. Specifically, individuals were classified as having baseline-consistent pigmentation (≤8% irregularity; see above) or elevated pigmentation (>8%), corresponding to the upper bound of variation observed in pre-outbreak irises. Differences in antibody reactivity between these groups were assessed using non-parametric tests (Wilcoxon rank-sum), as assumptions of normality were not met. 

To explore whether AIV exposure and iris pigmentation irregularities may affect physiological condition via impaired vision and reduced foraging efficiency, we tested for associations among body mass, antibody reactivity, and iris pigmentation irregularity. Raw body mass was used as a coarse proxy for condition, as no additional morphometric measurements were available to calculate a size-corrected body condition index. Associations between body mass and anti-NP antibody reactivity (S/N ratios), anti-H5 antibody reactivity (PI%) and iris pigmentation irregularity scores were assessed using Spearman’s rank correlation tests. For iris pigmentation irregularity, we evaluated relationships between body mass and both the iris with greater irregularity and the average irregularity score across both irises.

All figures were created using *ggplot2* (Wickham, 2016). We used a significance threshold of 0.05. Means are presented with standard deviation (±SD).

## Results

### Avian influenza screening and study overview

No mass mortality events were reported at Bonaventure Island during the 2023 breeding season. Iris pigment irregularities were not associated with obvious impaired behaviour. Although we did not directly assess visual function, birds with irregular irises exhibited normal flight, foraging, and alert responses before and during handling, and all individuals appeared in good condition. All birds sampled in 2023 (*n* = 65) tested negative for an active AIV infection via RT-PCR targeting the matrix and H5 gene. Of these, 40% tested positive for anti-NP antibodies. Among NP antibody-positive birds, 92% exhibited elevated iris pigmentation irregularities (>8%), while the remaining individuals fell within the baseline range (Table 1). Overall, 13 of 65 sampled birds (20%) tested positive for anti-H5 antibodies. Among these anti-H5 antibody–positive individuals, 69% exhibited elevated iris pigmentation irregularities and 31% exhibited baseline pigmentation (Table 1).

**Table 1:** Summary of AIV diagnostic results (anti-NP) antibody and anti-H5 and iris pigmentation irregularity categories in 65 northern gannets sampled at Bonaventure Island in 2023

**Test result**	***n* (%)**	**Baseline (≤8%)**	**Elevated (>8%)**
**Anti-NP antibody**			
Positive	26 (40%)	2 (8%)	24 (92%)
Negative	39 (60%)	33 (85%)	6 (15%)
**Anti-H5 antibody**			
Positive	13 (20%)	4 (31%)	9 (69%)
Negative	52 (80%)	31 (60%)	21 (40%)

### Pre-outbreak baseline iris pigmentation

The pre-outbreak baseline for iris pigmentation irregularities was 4.39 ± 1.84%, with a range of 0.22–8.02%. Birds sampled post-outbreak in 2023 that were visually classified in the field as having no obvious iris pigmentation irregularities (‘normal’ or non-black eyes) had a mean irregularity score of 2.52 ± 2.40%, with a range of 0.09–11.76%. Overall, these visually normal post-outbreak birds largely overlapped with the pre-outbreak baseline range, supporting the use of ~8% irregularity as an approximate upper threshold for baseline pigmentation.

### Categorical iris classes and antibody reactivity

**Figure 3:**
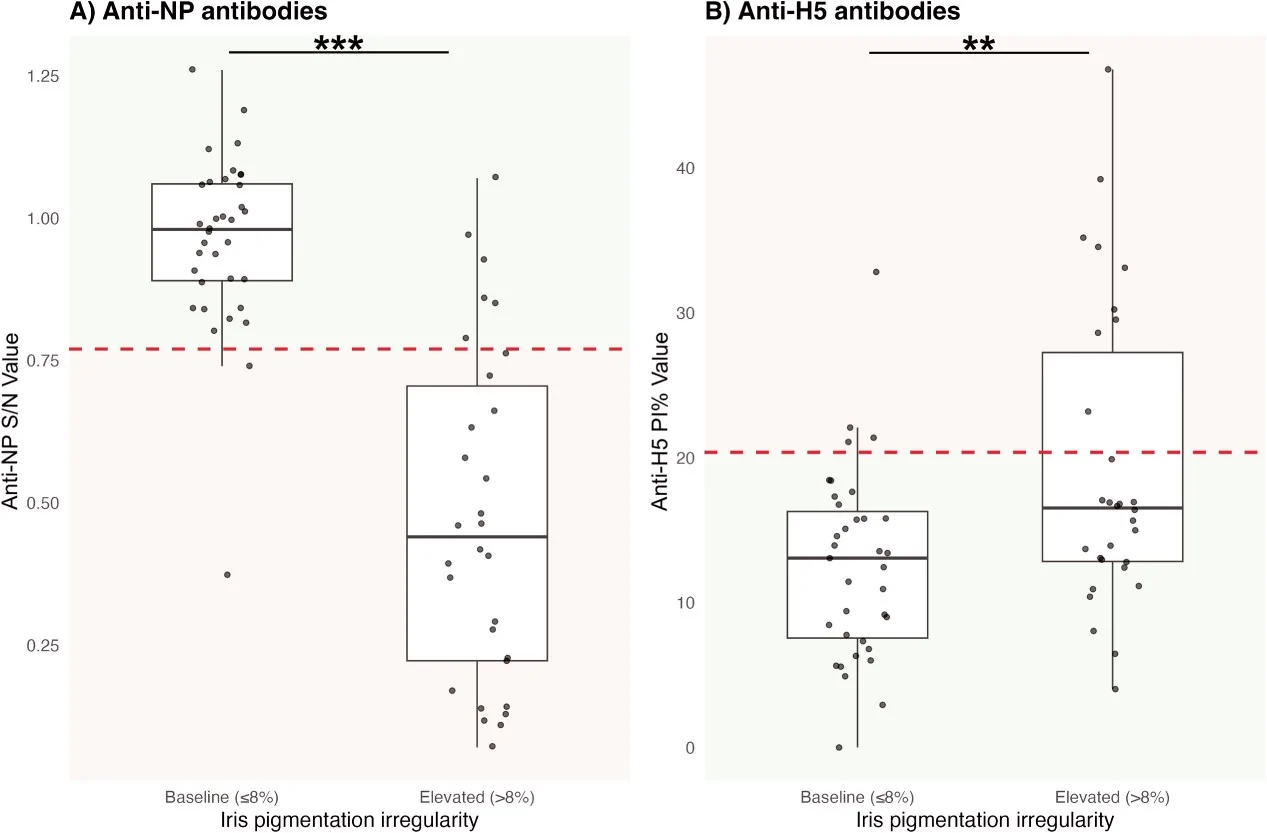
Boxplots of anti-NP antibody (A) and anti-H5 antibody (B) responses across two categories of iris pigmentation irregularity severity based on average iris irregularity per bird: low (<8%) and elevated (>8%). In (A), the dashed horizontal line indicates the optimized seropositivity threshold for the IDEXX anti-NP assay (S/N = 0.77; Giacinti *et al.*, 2025). Values below this threshold (red-shaded region) indicate seropositive results, whereas values above the threshold (green-shaded region) indicate seronegative results. In (B), the dashed horizontal line indicates the optimized seropositivity threshold for the NCFAD anti-H5 assay (PI% = 20.37; Giacinti *et al.*, 2025). Values above this threshold (red-shaded region) indicate seropositive results, whereas values below the threshold (green-shaded region) indicate seronegative results. Individual data points are shown as jittered black circles; boxplots show medians and interquartile ranges, with whiskers extending to 1.5× the interquartile range. ^***^ indicates *P* < 0.0001 and ^**^ indicates *P* < 0.05.

To assess whether iris pigmentation irregularity could provide a simplified field-based indicator of AIV exposure, birds were also grouped into baseline (≤8%) and elevated (>8%) categories of iris pigmentation irregularity. There was a significant difference in anti-NP antibody S/N ratios between birds with elevated and baseline iris pigmentation irregularity (Wilcoxon rank-sum test: *W* = 963, *P* < 0.001; Fig. 3). Birds with elevated iris irregularity exhibited lower S/N ratios, indicating higher anti-NP antibody reactivity.

**Figure 4:**
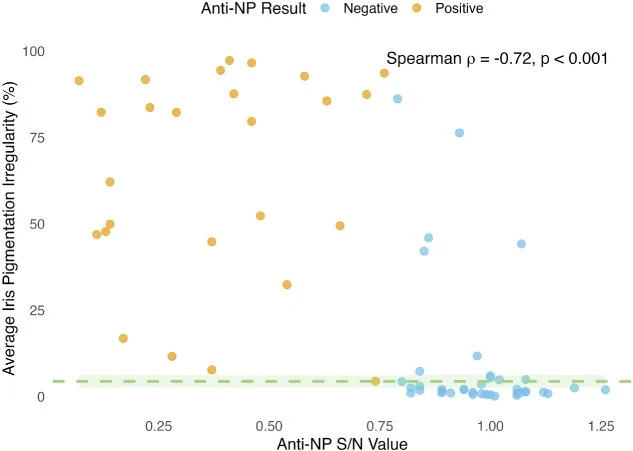
Correlation between anti-NP S/N ratios and iris pigmentation irregularity (%) for average irregularity across both irises. Points are coloured by anti-NP result interpretations (‘Positive’ or ‘Negative’). The dashed green line shows the average pre-outbreak (pre-2021) irregularity, with the shaded band representing ±1 SD. Note that lower S/N ratios correspond to stronger anti-NP antibody detection. An optimized threshold of S/N < 0.77 was used to classify samples as positive for anti-NP antibodies, based on threshold optimization for Nobuto strip testing at NWRC (Giacinti *et al.*, 2025).

**Figure 5:**
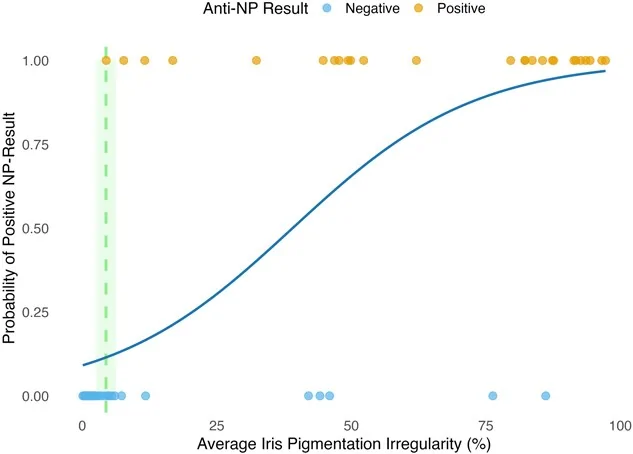
Logistic regression models predicting the probability of anti-NP antibody S/N ratio positivity based on average iris pigment irregularity. Points are coloured by test result interpretation (‘Positive’ or ‘Negative’) for each sample. The dashed light green line is average pre-outbreak (pre-2021) calculated irregularity, with the shaded band representing ±1 SD. The solid blue line indicates the predicted probability of seropositivity based on the fitted regression model.

A similar pattern was observed for anti-H5 antibody PI% values (*W* = 332.5, *P* = 0.012). Birds with elevated iris irregularity exhibited higher PI% values, indicating increased anti-H5 antibody reactivity (Fig. 3).

**Figure 6:**
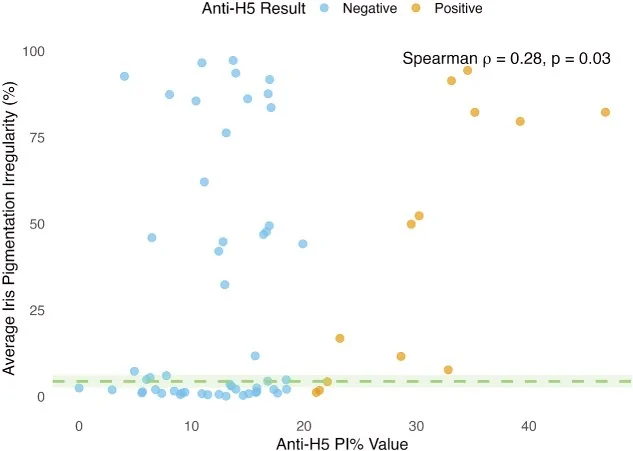
Correlation between anti-H5 PI% antibody detection and iris pigmentation irregularity (%) for average irregularity across both irises. Points are coloured by anti-H5 antibody result interpretations (‘Positive’ or ‘Negative’). The dashed light green line shows the average pre-outbreak (pre-2021) irregularity, with the shaded band representing ±1 SD. Note that higher PI values correspond to stronger anti-H5 antibody detection. A PI value ≥20.37% was considered positive, reflecting the optimized cut-off for Nobuto strips (Giacinti *et al.*, 2025).

Results were highly consistent across alternative iris metrics (Fig. S4). Anti-NP antibody S/N ratios were significantly lower in birds with elevated iris pigmentation irregularity when using the iris with greater irregularity (*W* = 970.5, *P* < 0.001), the left eye (*W* = 837.5, *P* < 0.001) and the right eye (*W* = 972.0, *P* < 0.001), indicating robust associations regardless of which eye was used to quantify pigmentation. Birds with elevated iris irregularity showed significantly higher PI% values when using the iris with greater irregularity (*W* = 358.5, *P* = 0.027) and the right eye (*W* = 333.5, *P* = 0.013), whereas the association was not significant when using the left eye (*W* = 368.5, *P* = 0.077), suggesting some sensitivity to measurement choice.

### Associations between iris pigmentation irregularity and anti-NP antibody reactivity

Across the full dataset, iris pigmentation irregularity was strongly negatively associated with anti-NP S/N ratios (*ρ* = −0.72, *P* < 0.001; Fig. 4), with higher irregularity scores corresponding to lower S/N ratios—i.e. more positive results for anti-NP antibodies. Comparable results were obtained when using the iris with greater irregularity and when analysing left and right eyes separately (Supplemental Fig. S1; left: *ρ* = −0.63; right: *ρ* = −0.65; all *P* < 0.001).

Consistent with this pattern, average iris pigmentation irregularity was a strong positive predictor of anti-NP antibody positivity in binomial models (*β* = 0.059 ± 0.013, *z* = 4.58, *P* < 0.001; Fig. 5; Fig. S3), explaining ~46% of model deviance. Specifically, for each 1% increase in average iris pigment irregularity, the odds of testing positive for anti-NP antibodies increased by ~6%. Similar patterns were observed when iris pigmentation irregularity was assessed separately for each eye (left: *β* = 0.044 ± 0.012, *z* = 3.82, *P* < 0.001; right: *β* = 0.047 ± 0.009, *z* = 5.03, *P* < 0.001).

### Associations between iris pigmentation irregularity and anti-H5 antibody reactivity

Average iris pigment irregularity also showed a significant positive correlation with anti-H5 antibody PI%, although the relationship was weaker (*ρ* = 0.27, *P* = 0.027; Fig. 6), with higher irregularity scores associated with higher PI% values—i.e. more positive results for anti-H5 antibodies. Results were again similar for all single-eye analyses (Fig. S2; left: *ρ* = 0.27, *P* = 0.031; right: *ρ* = 0.28, *P* = 0.024).

However, average iris pigment irregularity was not a significant predictor of anti-H5 antibody positivity (*β* = 0.01, *z* = 1.39, *P* = 0.17), and the model explained only ~3% of the deviance. Results were similar for single-eye analyses (left: *β* = 0.008, *z* = 1.04, *P* = 0.30; right: *β* = 0.011, *z* = 1.51, *P* = 0.13), with models explaining ~1.6% and ~3.6% of the deviance, respectively. This suggests that variation in iris pigmentation was not associated with the probability of testing positive for anti-H5 antibodies.

### Associations between iris pigmentation irregularity and antibody reactivity among seropositive individuals

To evaluate whether iris pigmentation variation reflected variation in anti-NP antibody reactivity among exposed individuals, analyses were restricted to seropositive birds. Among anti-NP antibody–positive birds, there was no significant association between S/N ratios and iris pigmentation irregularity (average: *ρ* = 0.15, *P* = 0.46; iris with greater irregularity: *ρ* = 0.32, *P* = 0.11; left eye: *ρ* = 0.07, *P* = 0.75; right eye: *ρ* = 0.11, *P* = 0.60). This indicates that variation in anti-NP reactivity does not scale with the extent of iris pigmentation change within AIV exposed individuals.

In contrast, among anti-H5 antibody–positive birds, iris pigmentation irregularity was strongly positively associated with anti-H5 antibody PI% values (average: *ρ* = 0.85, *P* < 0.001; iris with greater irregularity: *ρ* = 0.70, *P* = 0.01; left eye: *ρ* = 0.85, *P* < 0.001; right eye: *ρ* = 0.65, *P* = 0.020), suggesting that H5 subtype-specific antibody reactivity covaries with the degree of iris pigmentation irregularity among exposed birds.

### Body mass associations

We did not find any correlation between body mass and anti-NP antibody reactivity (S/N ratios) or anti-H5 antibody reactivity (PI%) (*ρ* = −0.13, *P* = 0.30; *ρ* = 0.23, *P* = 0.06, respectively). No significant association was detected between body mass and the iris with a greater irregularity score (*ρ* = 0.23, *P* = 0.06), nor between body mass and average irregularity score (*ρ* = 0.20, *P* = 0.12).

### Within-season temporal stability (2024)

Similar to 2023, there were no mass mortality events at the colony in 2024. Temporal assessment of seven individual gannets revealed overall stable iris pigmentation across the 2024 breeding season (Fig. S5). On average, the within-individual range in pigment irregularities was 4.6 ± 2.7%. Changes were not consistent in direction, four showed small decreases, while the remainder exhibited minor increases.

## Discussion

### Iris pigmentation irregularity as an indicator of AIV exposure

Building on Lane *et al.* (2024), who first documented iris pigmentation changes in gannets following HPAIV outbreaks, this study evaluates the utility of this visible, sublethal physiological change as a minimally invasive indicator of prior AIV infection. Following the 2022 HPAIV outbreak, iris pigmentation irregularities were associated with circulating anti-AIV antibodies, suggesting survival and recovery after infection (Lane *et al.*, 2024). Additional population-level evidence comes from post-outbreak shifts in yolk antibody profiles at Bonaventure Island, where increases in both anti-NP and anti-H5 antibody reactivity from 2022 to 2023 indicate elevated AIV exposure following the outbreak (McLaughlin *et al.*, 2025).

Whereas Lane *et al.* (2024) relied on categorical eye-colour classifications, we quantified iris pigmentation irregularity on a continuous scale and examined how variation relates to antibody detection. Using a larger dataset collected ~1 year after the 2022 HPAIV outbreak, we demonstrate that increased iris pigmentation irregularity is significantly associated with anti-NP antibody detection. Birds exhibiting greater departures from baseline iris pigmentation (ranging from traces of black spots to almost fully blackened irises) were more likely to test positive for anti-AIV antibodies, with this pattern driven primarily by anti-NP antibody positivity. Average iris pigmentation irregularity was also weakly positively correlated with anti-H5 antibody reactivity (PI%), with higher irregularity associated with higher PI% values. However, iris pigmentation irregularity was not a significant predictor of anti-H5 antibody positivity. These relationships indicate that iris pigmentation irregularity may function as a probabilistic indicator of prior AIV exposure, particularly as reflected by anti-NP antibody positivity, while the relationship with anti-H5 antibody reactivity appears weaker.

Consistent with Lane *et al.* (2024), our categorical analyses also showed higher anti-H5 antibody reactivity among birds with black irises, supporting the previously reported association between iris darkening and prior HPAIV exposure. However, our additional analyses using the continuous iris pigmentation irregularity score revealed a more nuanced relationship from those of Lane *et al.* (2024), who reported a strong correspondence between black irises and H5 seropositivity shortly after the 2022 outbreak. In contrast, we observed iris irregularities in some H5-antibody-negative birds, and H5 serostatus was not consistently predicted by pigmentation irregularity. This discrepancy likely reflects differences in sampling timing and antibody dynamics rather than the absence of prior AIV exposure. Lane *et al.* (2024) sampled birds immediately following the outbreak, whereas our samples were collected ~1 year later, when subtype-specific H5 antibodies may have waned below the threshold for positivity despite persistence of the visible iris phenotype. Differential waning rates between H5-specific and NP antibodies have previously been documented in wild birds (Giacinti *et al.*, 2024a), as well as specifically in ducks (Wight *et al.*, 2024), and reduced sensitivity associated with Nobuto strip sampling may further contribute to false-negative results on the H5 assay. In addition, individual variation in immune response and/or the duration of pigmentation persistence following infection may contribute to imperfect correspondence between iris phenotype and serostatus. H5-seropositive individuals that lacked visible iris pigmentation irregularities (4/13) could reflect variation in the development or persistence of the phenotype, although false-positive serological results cannot be excluded. Further investigation of these discordant cases would help clarify whether they represent true prior HPAIV exposure in the absence of persistent iris changes or limitations of serological classification. Consequently, iris pigmentation irregularities are best interpreted as indicators of H5 exposure rather than guaranteed markers of contemporaneous H5 antibody positivity.

Analyses of AIV antibody assay results indicated that birds with more pronounced iris irregularities were more likely to exhibit serological evidence of prior AIV exposure, characterized by lower S/N ratios (for anti-NP) and higher PI% values (for anti-H5). In practical terms, these metrics reflect the relative degree of antibody binding detected by the assay and were used here as indirect measures of prior viral exposure and survival, rather than indicators of current infection, antibody titre, or protective immunity. These analyses were intended to explore whether increasing iris irregularity corresponded with gradual changes in antibody reactivity rather than discrete diagnostic categories. When all individuals were considered together, the observed relationships suggest a biological continuum in which birds with relatively normal irises tended to show little or no serological evidence of prior AIV exposure, whereas birds with greater iris irregularity more often exhibited seropositivity and greater antibody reactivity. However, among seropositive individuals, the extent of iris irregularity was not associated with anti-NP antibody reactivity, whereas a positive association persisted for anti-H5 PI% values, suggesting that iris pigmentation variation primarily reflects exposure history (i.e. the likelihood that an individual was previously infected) rather than broad variation in antibody reactivity among exposed individuals. The continued association with H5-specific reactivity may indicate that iris pigmentation irregularity may capture additional biological variation. Potential explanations include differences in the timing, severity, or immunological consequences of prior H5 infection, although these possibilities could not be evaluated directly in the present study.

The mechanisms linking immune response to iris pigmentation changes remain unclear but may involve immune-mediated inflammation, direct or indirect effects of viral infection on ocular tissues, or broader systemic physiological responses associated with prior infection. Importantly, these processes are not necessarily expected to scale with antibody titre or ELISA reactivity but may instead reflect infection history and host response dynamics during or following exposure. Recent ophthalmological and histopathological investigations of gannets provide evidence for both inflammatory and melanocytic changes underlying abnormal iris pigmentation, including frequent signs of uveitis and increased melanin deposition within the iris (Fontaine *et al.*, 2026). These findings provide a potential mechanistic link between infection and the observed iris phenotype and are consistent with the association between abnormal pigmentation and H5 seropositivity reported in the same study. While ocular manifestations associated with HPAIV have been reported in other species (Brown *et al.*, 2008; Yamamoto *et al.*, 2016; Alexandrou *et al.*, 2025), similar ocular pathology has also been observed in response to other infectious agents, such as retinal lesions associated with *Toxoplasma gondii* infection in birds (Williams *et al.*, 2001). Thus, although existing evidence supports a plausible biological basis for infection-associated ocular change, the specific processes leading to the development, persistence and variation of iris pigmentation irregularities in gannets remain unresolved. Future research should further investigate the tissue- and cellular-level processes responsible for these pigmentation changes and assess whether they result from direct effects of infection on ocular tissues or broader physiological responses to infection. Additional work is also needed to understand why the extent of pigmentation irregularity varies among individuals, including the potential roles of host immune responses, infection severity, exposure dose or route, and variation among infecting viral strains.

### Stability and temporal dynamics of iris pigmentation irregularity

Within-individual variation over the 2024 breeding season was low (mean range = 4.6 ± 2.7%), indicating that once established, iris pigmentation irregularities were largely stable over several months. Repeated field photographs collected over 6–8 weeks showed little change in pigmentation patterns, suggesting that these irregularities persist for at least part of the breeding season rather than representing a transient clinical sign. However, these images were obtained from greater distances (2–5 m) than the standardized close-up photographs used for quantitative scoring and were therefore less sensitive to subtle pigmentation changes. Representative images from the repeated-measures dataset are provided in Fig. S5.

Despite limited within-season variability, substantial variation in iris pigmentation irregularity was observed among individuals. The factors underlying this variation remain unclear but may include differences in infection history, infection severity, or individual host responses. Since all data were collected during the 2023 and 2024 seasons, well after the peak 2022 HPAIV outbreak in gannets, the observed within-season stability of iris pigmentation irregularities suggests that these changes can persist for many months following infection. However, the extent to which pigmentation patterns reflect a single historical infection versus subsequent exposures remains uncertain, as continued or renewed exposure during wintering or pre-breeding periods cannot be excluded. Supporting this possibility, yolk antibody data from the same colony indicate the presence of AIV-related antibodies in 2023 eggs, consistent with either re-exposure or prolonged AIV antibody persistence following the 2022 outbreak (McLaughlin *et al.*, 2025).

AIV antibody persistence remains poorly understood in wild birds, but available evidence indicates substantial variation among antibody types and taxa. For example, NP antibodies persisted longer than H5 antibodies in an urban duck population monitored before and during an H5N1 outbreak (Wight *et al.*, 2024; Giacinti *et al.*, 2024a), highlighting differential waning rates and the potential for antibodies to remain detectable months to years after exposure. However, repeated exposures to AIV may further influence antibody persistence, complicating the interpretation of exposure histories. Comparable data are lacking for long-lived seabirds, but similar antibody persistence is biologically plausible.

In this context, iris pigmentation irregularities may function as a complementary biomarker that integrates exposure history over longer timescales than seropositivity alone. If iris changes persist after subtype-specific antibodies (e.g. H5) decline below positivity thresholds, they could provide evidence of prior infection even after serological signals have waned. Conversely, if antibody reactivity persists over multiple years, iris irregularities may reflect cumulative or repeated exposure rather than recent infection. Consequently, while our findings demonstrate a clear association between iris pigmentation irregularities and AIV exposure, we cannot distinguish whether observed antibody positivity and pigmentation changes reflect recent infection, re-exposure, or persistence of antibodies following the 2022 outbreak. Notably, no HPAIV-attributable mortality events were reported at the colony in 2023, indicating that substantial on-colony mortality was unlikely during that period; however, exposure history during time spent away from the colony remains unknown. Resolving these temporal dynamics will require studies combining repeated standardized iris imaging with serological and virological sampling across multiple years.

### Implications for field detection and population monitoring

While mortality has been the most visible consequence of H5 HPAIV clade 2.3.4.4b outbreaks in many seabird populations globally, sublethal effects are increasingly recognized as critical for understanding the full impact of infection on wild populations. Sublethal consequences of AIV infections in birds have been associated with reduced breeding propensity and movement (Gamble, 2023), delayed migration (Teitelbaum *et al.,* 2023a, 2023b), lower body mass (Kuiken, 2013), and increased contaminant burdens (Teitelbaum *et al.*, 2022). However, detailed, individual-level evidence of sublethal effects in free-ranging seabirds remains scarce, particularly in the aftermath of large-scale HPAIV outbreaks. In this context, our study provides evidence that a visible, non-lethal phenotypic change in gannets is associated with prior AIV exposure. Because iris pigmentation irregularities are readily observable in the field, they represent a low-cost, practical, and minimally invasive complement to serological monitoring. Our categorical grouping approach was used to evaluate whether broad visual assessments capture the same exposure signal identified in continuous measurements. Across analyses, birds with greater iris irregularity, whether quantified continuously or classified using a threshold-based approach, consistently showed a higher likelihood of AIV antibody detection, particularly for anti-NP antibodies (Fig. 3). Importantly, this pattern was not restricted to extreme ‘black-eyed’ phenotypes, suggesting that the exposure signal is expressed across a gradient of visible pigmentation rather than as a discrete category.

Anti-NP antibody positivity (a general marker of past AIV exposure) increased with iris pigmentation irregularity. Individuals with ~40% irregularity had a >50% probability of testing positive for anti-NP antibodies, rising to ~77% at 60%, ~86% at 70% and >90% at 80% (Fig. 5). Moderate to high levels of iris pigmentation irregularity were absent from pre-outbreak control data, supporting the inference that pigmentation exceeding the historical baseline range is consistent with AIV exposure-associated phenotypic change. Based on this distribution, we defined >8% irregularity as the upper bound of historical variation, separating baseline-consistent from elevated pigmentation. The probability values across higher irregularity levels describe a continuous exposure–response relationship rather than diagnostic thresholds. Together, these results suggest that even moderate darkening (>8%) may serve as a practical indicator of previous AIV exposure in gannets.

Iris pigmentation irregularities can be quantified most reliably using standardized photographs collected during banding or nest checks. Image-based scoring provides an objective, continuous measure of irregularity and is relatively fast, taking 5–10 minutes per iris with Photoshop, and could be further streamlined using free, open-source software such as ImageJ (https://imagej.net/ij/). While direct visual scoring is not equivalent to this quantitative approach, a simplified threshold-based field assessment may provide a practical alternative for rapid screening. For example, as demonstrated here, birds could be classified as exhibiting either baseline or elevated iris irregularity using approximate visual thresholds informed by pre-outbreak variation. Such categorical scoring allows rapid, low-impact screening when photography is not feasible, although image-based scoring remains preferable for population-level monitoring, threshold validation, and comparative studies.

Practical implementation requires standardized protocols and trained observers to ensure repeatability, as detection accuracy may be influenced by lighting, viewing distance and observer variability. Close-up photography improves measurement precision but may require temporary handling or close approach, which can limit feasibility in dense colonies. Accordingly, iris pigmentation scoring is best interpreted alongside traditional viral surveillance and serological data, rather than as a stand-alone diagnostic tool. In this framework, serology remains the primary laboratory method for investigating AIV exposure, whereas iris scoring offers a rapid first-pass indicator to prioritize sampling effort and estimate exposure prevalence when resources are limited, providing a practical tool for population-level assessment.

### Sublethal effects and fitness consequences in surviving individuals

Birds with irregular irises exhibited normal flight, foraging and behavioural responses during field observations, and iris pigmentation irregularity was not associated with variation in body mass among individuals. While only 13% of birds with irregular pigmentation were observed on active nests with chicks compared with 80% of birds with regular irises, all chick-rearing birds with irregular irises engaged in routine breeding behaviours, including nest attendance, partner exchange, and chick provisioning. The lower proportion of irregular iris birds on active nests may partly reflect the timing and context of sampling, which occurred late in the breeding season. Consequently, we cannot determine whether irregular iris individuals had attempted breeding earlier in the season. Survivors of the 2022 outbreak may also have experienced partner loss or delayed re-pairing, reducing breeding participation independently of physiological condition. The lower representation of birds with irregular irises among active breeders may therefore reflect factors unrelated to current physiological condition.

Context from previous work supports this interpretation. Lewis *et al.* (2025) found that gannets with irregular iris pigmentation had similar breeding success to unaffected individuals at two UK colonies, and Ponchon *et al.* (2026) reported that 1 year after the 2022 H5N1 outbreak, irregular iris pigmentation gannets at the Rouzic colony in France did not differ in at-sea foraging behaviour or habitat use, with breeding success comparable to or higher than pre-outbreak years. Together, these studies suggest that surviving gannets exhibiting iris pigmentation irregularities can maintain breeding and foraging performance comparable to unaffected individuals.

The lack of association between iris pigmentation irregularities and body mass suggests that the extent of iris pigmentation change was not associated with variation in body mass among individuals at the time of sampling. However, body mass alone may not capture more subtle effects on physiological condition or energetic status. Morphological markers have been shown to persist following infection with other pathogens in wild avian populations. For example, bill deformities linked to avian keratin disorder (Van Hemert *et al.*, 2012, 2013; Zylberberg *et al.*, 2018) and ocular swelling from *Mycoplasma gallisepticum* (a prokaryote) infections (Thomason *et al.*, 2017) in birds. In contrast, few studies have identified subtle, non-debilitating and quantifiable morphological traits, like iris pigmentation irregularities, that could reliably persist across time and reflect prior infection in wild populations. This approach may be particularly valuable in pale-iris species, where pigmentation anomalies are more easily detected, and in contexts where repeated handling or blood sampling is impractical, such as large or dense breeding colonies, remote field sites, or situations requiring rapid non-invasive population screening. However, we emphasize that the >8% cut-off used to distinguish baseline-consistent from elevated pigmentation should be viewed as provisional, pending further validation.

Observations of repeatedly monitored individuals in our study further support this conclusion: ~71% of nests belonging to birds with irregular iris pigmentation produced chicks that survived to late summer, despite mean iris pigmentation irregularity scores above 36%. This level of reproductive success closely matches colony-level productivity in 2024, when breeding success reached 70.8% per occupied territory (81.9% hatching success and 90.1% fledging success). However, breeding success at this colony has been highly variable in recent years, with some seasons exhibiting substantially lower productivity (Pelletier *et al.*, 2023). Together with prior work, these findings further indicate that iris pigmentation irregularities do not necessarily imply reduced condition or reproductive performance in surviving individuals.

### Limitations and future directions

While our findings are consistent with a post-outbreak effect, we cannot conclusively determine whether iris pigmentation irregularities were driven specifically by the H5 HPAIV clade 2.3.4.4b, or another AIV strain, due to limitations in serotype resolution and sample size. Anti-NP antibodies provide evidence of general AIV exposure, while anti-H5 antibodies indicate exposure to an H5 subtype but not necessarily H5 HPAIV. However, given the colony’s negative status prior to 2022 (Lane *et al.*, 2024), the confirmed detection of H5 HPAIV during large-scale mortality events, and the lack of evidence for concurrent circulation of low-path H5 or other AIV strains at the time of this study, we assume that most antibody-positive individuals were exposed to H5 HPAIV. We acknowledge the limitations of this assumption and recommend further virological and serological surveillance to refine this interpretation.

Differences between NP and H5 results may also reflect variation in antibody persistence, as NP antibodies are known to remain detectable longer than subtype-specific antibodies in some wild bird populations (Giacinti *et al.*, 2024a; Wight *et al.*, 2024). Methodological constraints may have further influenced detection sensitivity. Nobuto filter paper samples, particularly when stored at room temperature, are not considered the gold standard for antibody detection compared to serum obtained via venipuncture and centrifugation (Giacinti *et al.*, 2025). Filter paper sampling was associated with reduced sensitivity relative to serum, particularly for H5 antibodies, even when assay thresholds are adjusted to account for expected signal attenuation (Giacinti *et al.*, 2025). Reduced assay sensitivity, variable elution efficiency, and potential antibody degradation during storage may therefore have contributed to false negatives or diminished quantitative resolution, although steps were taken to minimize these.

Iris pigmentation changes may also not be specific to AIV. Similar alterations could, in theory, arise from other infections or inflammatory processes that induce local immune responses or melanin deposition in the iris. Although we found no evidence of concurrent disease outbreaks at the colony, we cannot fully exclude alternative causes of inflammation or pigment change. In addition, our analyses did not fully account for potential confounding variables such as sex, age, or size-corrected body condition, as only body mass was available as a coarse proxy for condition. Future studies, where possible, should incorporate additional covariates and morphometric measurements to clarify their influence on immune response, disease recovery, and pigmentation patterns.

A further limitation concerns photographic methodology and measurement repeatability. Small changes in eye–camera angle, distance, shading, or illumination can influence the apparent proportion of dark pigmentation, particularly for subtle irregularities (<5% of the iris). Although pigmentation was quantified as a proportional metric to reduce sensitivity to moderate changes in iris shape or size, minor variation in viewing angle and lighting is unavoidable under field conditions. The minimal detectable level of pigmentation irregularity was ~2–5%, below which misclassification is more likely. Both within-observer repeatability (*R*^2^ = 0.993) and inter-observer reproducibility (*R*^2^ = 0.992) were high, indicating that the image-processing workflow yielded highly consistent estimates when applied repeatedly and by different analysts. Future work should nonetheless incorporate standardized image acquisition protocols and repeated imaging from comparable viewing angles to better quantify methodological uncertainty, although implementing such standardization in field conditions may be challenging due to variability in lighting, bird behaviour, and observer constraints.

The potential for iris pigmentation irregularity to change over time, whether via recovery or repeated exposures, presents both a strength and a challenge. While it may function as a dynamic biomarker, repeated infections could complicate interpretation. Thus, despite the promise of this approach, we caution that iris pigmentation may lose diagnostic value with repeated exposure. It remains unclear how multiple infections would influence iris changes, whether through progressive pigmentation, stabilization, or reversal. Also, once a bird develops iris irregularities, detecting subsequent exposures may be more difficult. We recommend that future field efforts include systematic annual iris photography or scoring of both eyes to build robust individual-level infection histories.

A further limitation of our study is the absence of repeated measurements of the same individuals across multiple years. Consequently, we cannot determine whether iris pigmentation irregularities persist long-term, whether they change with repeated exposures, or how antibody reactivity and iris changes co-vary over successive breeding seasons. While our within-season observations in 2024 indicate that iris irregularities remain relatively stable over several months, multi-year longitudinal data are needed to confirm the temporal stability of this trait and its potential as a reliable biomarker of prior AIV exposure. Importantly, such work is highly feasible in long-term monitored colonies where birds are permanently marked, as repeated standardized imaging of known individuals across breeding seasons could directly test persistence, progression, or reversal of pigmentation changes through time.

## Conclusion

By quantifying iris pigmentation irregularities at the population level, researchers could gain insight into several eco-epidemiological patterns, including (i) the relative prevalence of prior AIV exposure within a colony, (ii) variation in the occurrence of exposure-associated phenotypes among surviving individuals following outbreak events, (iii) spatial patterns of exposure across colonies or regions, and (iv) temporal changes in exposure history through repeated monitoring of individuals across seasons. Combined with serological and reproductive data, these measurements could provide insight into disease resilience, herd immunity, and the potential for sublethal effects to influence population dynamics, offering a practical complement to traditional viral surveillance. This may be a particularly useful set of tools for colonies that are visited and monitored less frequently. Visual biomarkers such as iris pigmentation changes are particularly valuable because, once thresholds are established, they enable minimally invasive, cost-effective, long-term surveillance of disease dynamics in free-ranging wildlife. Our findings suggest that iris pigmentation irregularity represents a scalable biomarker of prior HPAIV exposure in gannets, expanding the practical toolkit available for wildlife disease ecology and post-outbreak population monitoring. Larger, longitudinal studies will be required to assess potential subtle fitness consequences and to refine the reliability of iris pigmentation assessment as a scalable, field-based tool for long-term population monitoring.

## Supplementary Material

Web_Material_coag069

## Data Availability

The individual-level data supporting the findings of this study are provided in the supplementary material accompanying this article (Supplementary material).
